# Penicillin Allergy Delabeling: Provider Knowledge, Practice Patterns, and Barriers Among Internal Medicine Residents in a Large Urban Community Teaching Hospital

**DOI:** 10.7759/cureus.97928

**Published:** 2025-11-27

**Authors:** Tatevik Aloyan, Dinara Salimova, Evgenii Antonov, Gopika Chandra, Kris McGrath

**Affiliations:** 1 Internal Medicine, Ascension Saint Joseph Hospital, Chicago, USA; 2 Internal Medicine, Presbyterian Hospital, Albuquerque, USA; 3 Allergy and Immunology, Ascension Saint Joseph Hospital, Chicago, USA

**Keywords:** allergy delabeling, antimicrobial stewardship, beta-lactam allergy, clinical decision-making, electronic health records, pen-fast, penicillin allergy, quality improvement, resident education

## Abstract

Background: Penicillin allergy is the most frequently reported drug allergy, yet up to 90% of labels are inaccurate. These erroneous labels contribute to suboptimal antibiotic use, higher healthcare costs, and increased antimicrobial resistance. Despite growing evidence supporting delabeling, provider hesitancy and knowledge gaps persist.

Objective: The purpose of this study is to evaluate the awareness, confidence, and clinical decision-making of internal medicine residents regarding penicillin allergy delabeling within a large community hospital setting. Specifically, we seek to identify existing knowledge gaps, provider attitudes, and institutional barriers that may hinder appropriate delabeling efforts.

Methods: A cross-sectional, anonymous electronic survey was administered to internal medicine residents at Ascension Saint Joseph Hospital in Chicago. The survey included questions on allergy knowledge, delabeling practices, confidence in risk stratification, and perceived barriers. Responses were analyzed using descriptive statistics and chi-square tests.

Results: Of 66 residents invited, 42.4% (n = 28) responded. While 60.7% (n = 17) reported self-perceived knowledge on how to stratify beta-lactam allergy risk, only 12% (n = 2) felt confident in doing so. Familiarity with the PEN-FAST score increased with training level (postgraduate year (PGY)1: 0%; PGY3: 77%; p = 0.017). Half of the respondents had removed a penicillin allergy label, with significant differences by training level (p *= *0.048). Major barriers included lack of institutional protocols (n = 23, 82.14%), insufficient knowledge (n = 18, 64.3%), and time constraints (n = 12, 42.86%).

Conclusions: Significant gaps exist in residents' knowledge and confidence regarding the delabeling of beta-lactam allergies. Lack of standardized training and protocols further hinders effective implementation. Structured educational interventions and institutional support are needed to promote safe and evidence-based delabeling practices as part of antimicrobial stewardship.

## Introduction

Penicillin allergy is the most frequently documented drug hypersensitivity; however, its diagnosis remains largely inaccurate [1-4]. Symptoms commonly reported as allergic are often known adverse effects, rashes with infectious origin, or a result of viral/antibiotic interactions, resulting in patients being incorrectly labeled as allergic [1-4]. The penicillin allergy labels created during childhood frequently persist into adulthood without reassessment [1,4]. It has been shown repeatedly that the penicillin allergy label is not benign. It has significant clinical consequences, including prolonged hospital stays, limited antibiotic options, higher healthcare costs, and increased risk of antimicrobial resistance. Additionally, inappropriate labeling is linked to adverse drug reactions, suboptimal antibiotic selection, and even increased all-cause mortality [2,3,5].

Despite evidence and guidelines that removing erroneous beta-lactam allergy labels improves patient outcomes, many healthcare providers remain hesitant to do so. A thorough understanding of current clinical approaches, as well as the safety and benefits of beta-lactam allergy delabeling, is essential to improving these efforts. Recently, new tools such as the PEN-FAST decision rule have been introduced to help stratify the risk of beta-lactam allergies and identify patients who may or may not require formal allergy evaluation [5-7].

Objective

The primary purpose of this study is to evaluate the awareness, confidence, and clinical decision-making of internal medicine residents regarding penicillin allergy delabeling within a community hospital setting. The secondary aim is to identify possible barriers and modifiable system-level factors that may hinder appropriate delabeling efforts. This survey is part of a quality improvement project aiming to enhance the evidence-based penicillin allergy delabeling practices in our hospital, ultimately contributing to a broader goal of high-value patient care and antimicrobial stewardship. 

## Materials and methods

This study was an observational, cross-sectional, single-center survey conducted among internal medicine residents in a residency training program in Chicago, Illinois. The electronic medical records software was EPIC (Epic Systems Corporation, Verona, WI). The full survey instrument is provided in the supplementary appendix. The questionnaire was developed by the study team based on existing literature and reviewed for content clarity. The survey consisted of eight questions assessing respondents’ level of training, knowledge of penicillin allergy, and self-reported management practices. The questions were presented in multiple-choice formats with options for a comment. All residents were invited to participate voluntarily via an electronic survey anonymously and were instructed to refrain from using external resources when answering the questions. The research protocol was reviewed by the Ascension Institutional Review Board (IRB Study ID: RIL20250009) and was determined to be exempt from IRB approval.

Outcome measures and statistical analysis

Descriptive statistics were used to characterize the study population, as well as residents’ knowledge and practice patterns. A two-tailed chi-square test was used to compare survey responses and proportions across different training levels [8]. Each survey question was analyzed separately for each academic year for comparison. The MedCalc software was used for the statistical analysis, and MS Excel (Microsoft Corporation, Redmond, Washington, United States) was used for the statistical analysis and visualization.

## Results

**Figure 1:**
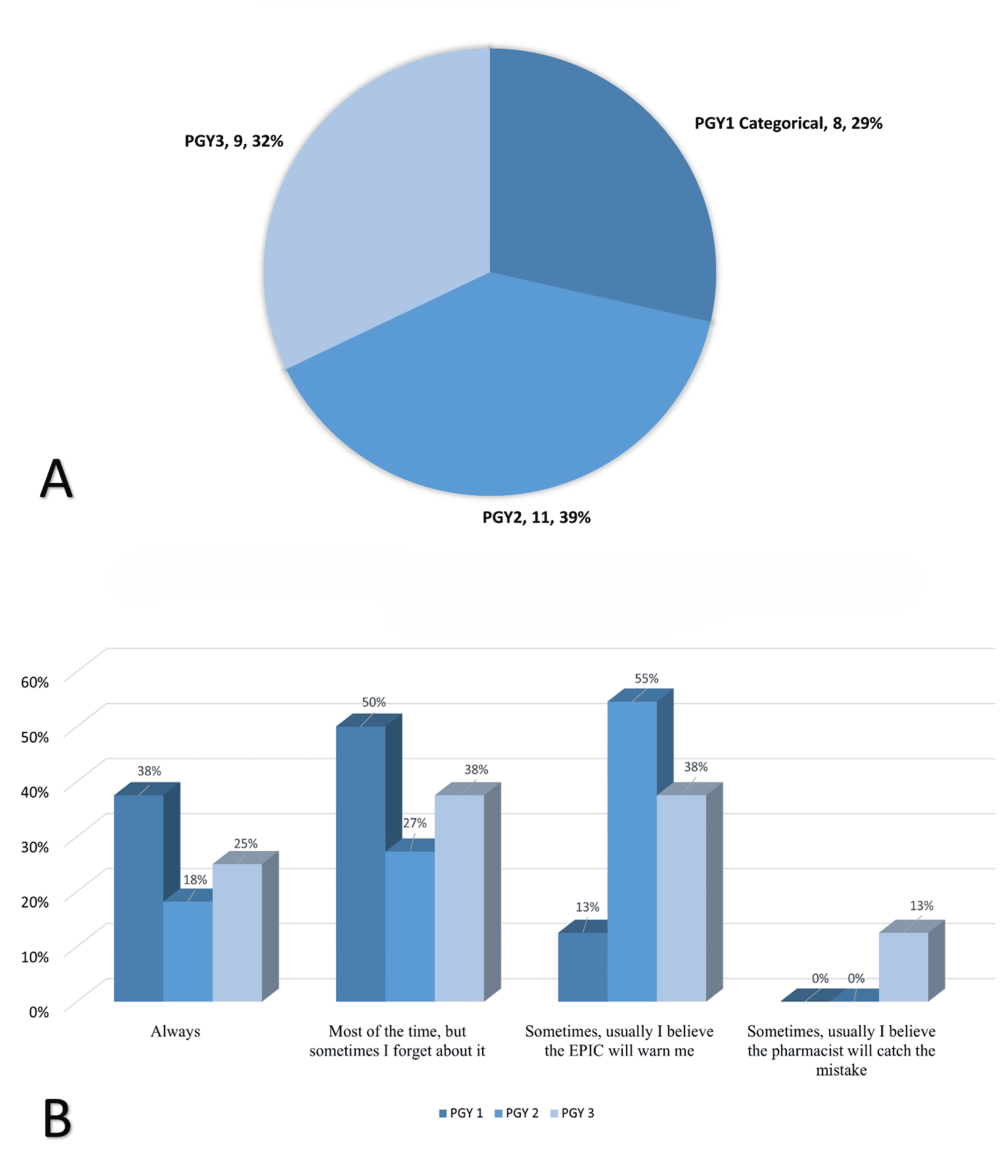
Resident training levels and frequency of allergy history review prior to antibiotic prescribing PGY: postgraduate year (A) Which residency year are you in? Response: PGY1 (n = 8; 29%), PGY2 (n = 11; 39%), PGY3 (n = 9; 32%) (B) How often do you review a patient’s allergy history in EPIC before prescribing antibiotics? (p = 0.44, chi-square test). Response: Always (PGY1: 38%, PGY2: 18%, PGY3: 25%). Most of the time, but sometimes I forget (PGY1: 50%, PGY2: 27%, PGY3: 38%). Sometimes, usually, I believe EPIC will warn me (PGY1: 13%, PGY2: 55%, PGY3: 38%). Sometimes, usually I believe the pharmacist will catch the mistake (PGY1: 0%, PGY2: 0%, PGY3: 13%)

A total of 66 internal medicine residents across all training years were invited to participate in the study, with 28 (42.4%) completing the survey. Among the respondents, eight residents (28.57%) were postgraduate year 1 (PGY1), 11 (39.29%) were PGY2, and nine (32.14%) were PGY3 (Figure 1A).

When asked how often they review a patient’s allergy history in EPIC before prescribing antibiotics, PGY1 residents were more likely to check the allergy section (n = 7, 87.5%), whereas senior residents were relatively more reliant on the electronic medical record (EMR) system to provide warnings (n = 10, 50%) (Figure 1B). However, this difference was not statistically significant (p = 0.44).

**Figure 2:**
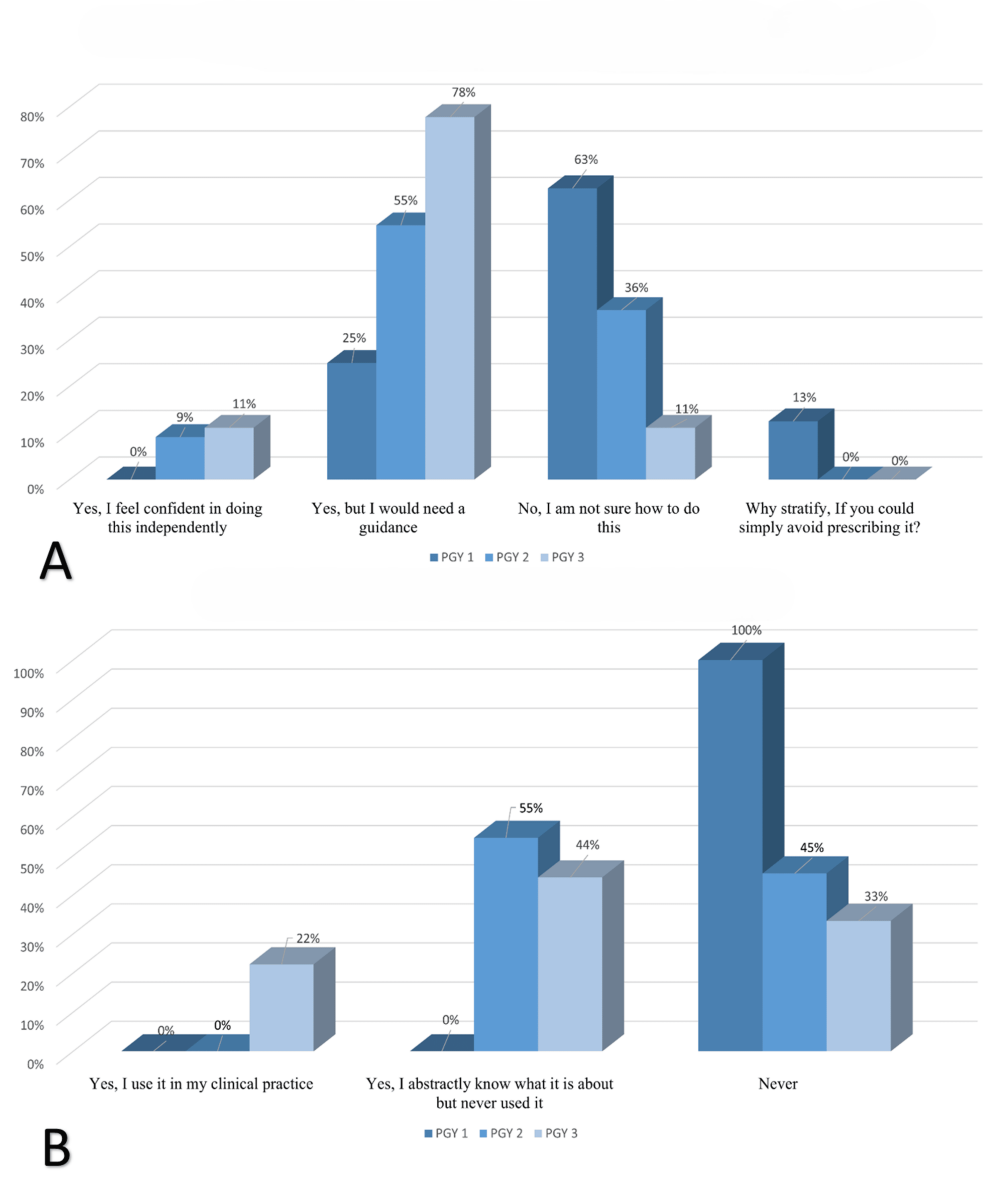
Knowledge and confidence in beta-lactam allergy risk stratification (A) Do you know how to stratify a patient’s beta-lactam allergy risk (e.g., low-risk vs. high-risk)? (p = 0.19, chi-square test). Responses: Yes, I feel confident in doing this independently (PGY1: 0%, PGY2: 9%, PGY3: 11%). Yes, but I would need guidance (PGY1: 25%, PGY2: 55%, PGY3: 78%). No, I am not sure how to do this (PGY1: 63%, PGY2: 36%, PGY3: 11%). Why stratify if you could simply avoid prescribing it? (PGY1: 13%, PGY2: 0%, PGY3: 0%). (B) Have you ever heard of the PEN-FAST score? (p = 0.017, chi-square test). Response: Yes, I use it in my clinical practice (PGY1: 0%, PGY2: 0%, PGY3: 22%). Yes, I abstractly know what it is about but never used it (PGY1: 0%, PGY2: 55%, PGY3: 44%). Never (PGY1: 100%, PGY2: 45%, PGY3: 33%)

Regarding their self-reported ability to stratify a patient’s beta-lactam allergy risk, 60.7% (n = 17) of respondents answered positively. However, only 12% (n = 2) felt confident doing so, while the remaining 88% (n = 15) would still require guidance to act (Figure 2A). Although senior residents were more likely to answer positively, the difference was not statistically significant (p = 0.19).

Familiarity with the PEN-FAST score also varied by training level. None of the interns completing the survey had heard of it, while 55% (n = 6) of PGY2 and 77% (n = 6) of PGY3 residents were aware of it (Figure 2B). Furthermore, 22% (n = 2) of PGY3 residents had applied it in clinical practice, revealing a statistically significant difference across training levels (p = 0.017).

**Figure 3:**
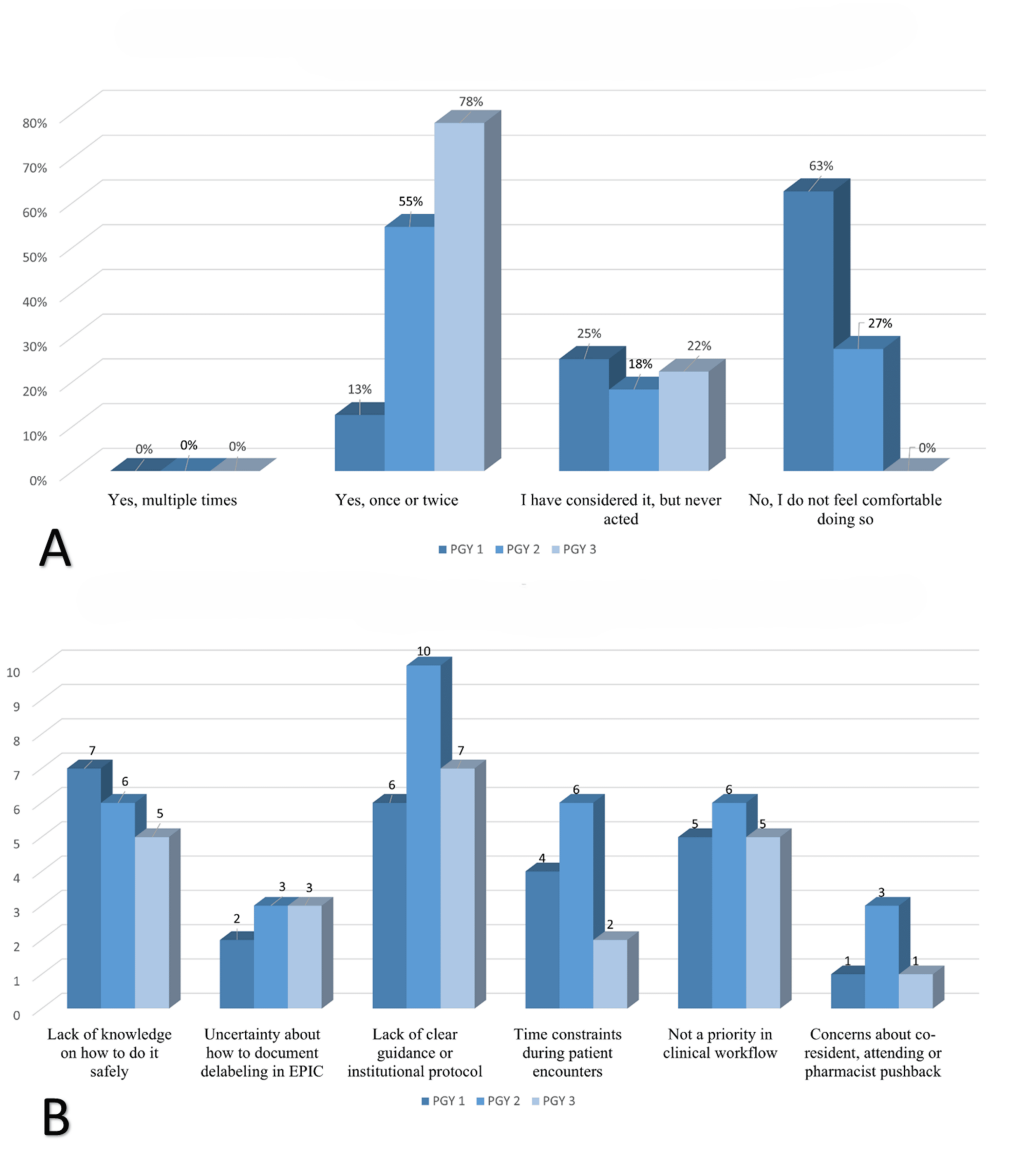
Experience with penicillin allergy delabeling and perceived barriers (A) Have you ever delabeled a patient’s penicillin/beta-lactam allergy in EPIC? (p = 0.048, chi-square test). Response: Yes, multiple times (PGY1: 0%, PGY2: 0%, PGY3: 0%). Yes, once or twice (PGY1: 13%, PGY2: 55%, PGY3: 78%). I have considered it, but never acted (PGY1: 25%, PGY2: 18%, PGY3: 22%). No, I do not feel comfortable doing so (PGY1: 63%, PGY2: 27%, PGY3: 0%). (B) What are the biggest barriers to delabeling beta-lactam allergies in your practice? Response: Lack of knowledge on how to do it safely (PGY1: n = 7, PGY2: n = 6, PGY3: n = 5). Uncertainty about how to document delabeling in EPIC (PGY1: n = 2, PGY2: n = 3, PGY3: n = 3). Lack of clear guidance or institutional protocol (PGY1: n = 6, PGY2: n = 10, PGY3: 7). Time constraints during patient encounters (PGY1: n = 4, PGY2: n = 6, PGY3: n = 2). Not a priority in clinical workflow (PGY1: n = 5, PGY2: n = 6, PGY3: n = 5). Concerns about co-resident, attending, or pharmacist pushback (PGY1: n = 1, PGY2: n = 3, PGY3: n = 1)

Half of the respondents (n = 14, 50%) reported having removed a penicillin allergy label at least once or twice, whereas the remaining half had never done so, though some had considered it (Figure 3A). A statistically significant difference was observed between senior residents and interns in delabeling experience, with 65% (n = 13) of senior residents having performed delabeling compared to only 12.5% (n = 1) of interns (p = 0.048).

The most frequently reported barriers to delabeling beta-lactam allergies were the lack of clear guidance or institutional protocol (n = 23, 82.14%), insufficient knowledge of how to perform delabeling safely (n = 18, 64.3%), and the perception that delabeling was not a clinical priority (n = 16, 57.14%) (Figure 3B). Other barriers included time constraints during patient encounters (n = 12, 42.86%), uncertainty regarding documentation in EPIC (n = 8, 28.57%), and concerns about resistance from co-residents, attending physicians, or pharmacists (n = 5, 17.86%).

**Figure 4:**
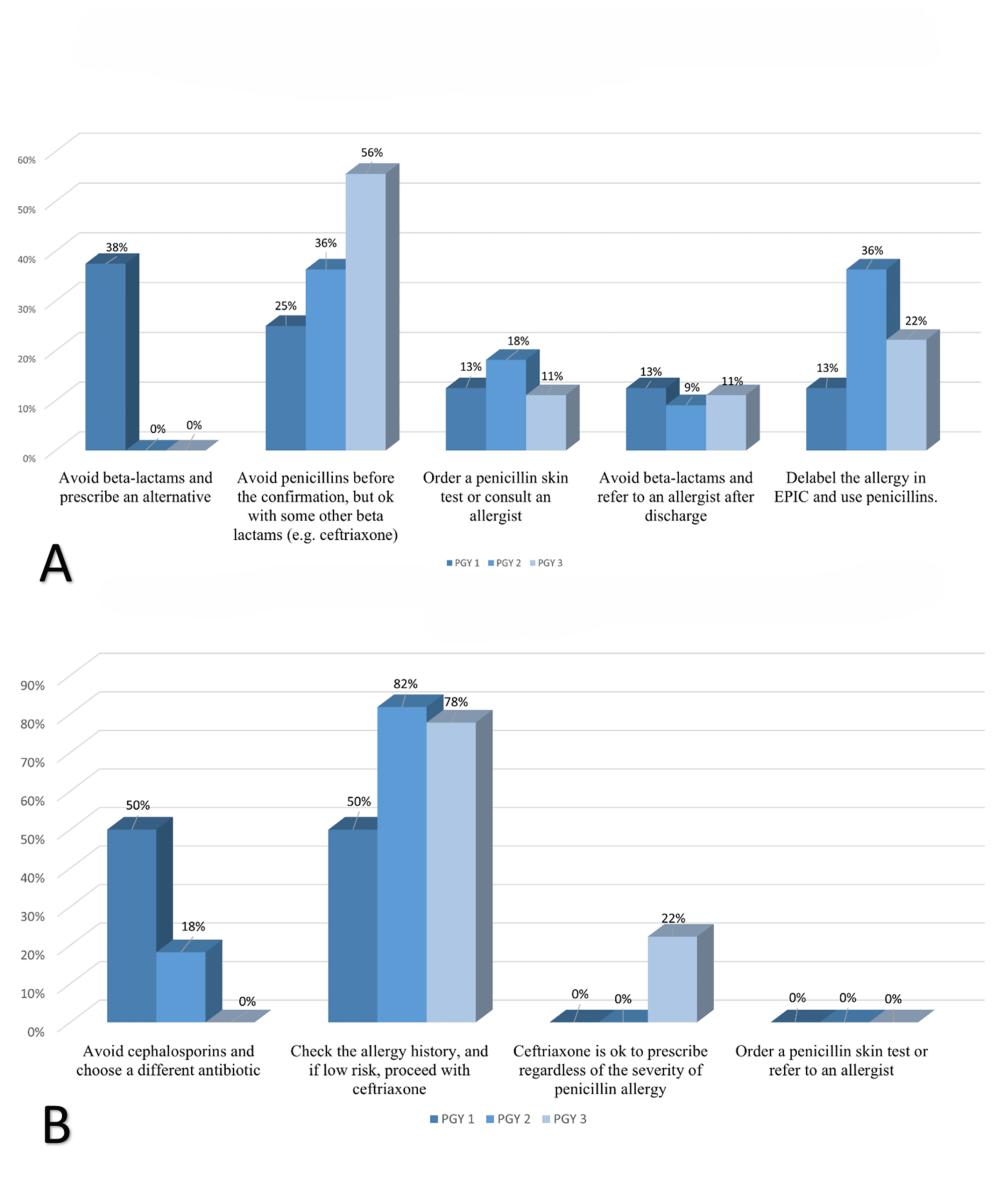
Resident responses to clinical vignettes assessing allergy-related decision-making (A) Case 1: Your patient has a documented penicillin allergy in EPIC but reports that they had a mild rash as a child (20y ago) with no other symptoms. How would you proceed? (p = 0.27, chi-square test). Response: Avoid beta-lactams and prescribe an alternative (PGY1: 38%, PGY2: 0%, PGY3: 0%). Avoid penicillins before confirmation, but use some other beta-lactams (e.g., ceftriaxone) (PGY1: 25%, PGY2: 36%, PGY3: 56%). Order a penicillin skin test or consult an allergist (PGY1: 13%, PGY2: 18%, PGY3: 11%). Avoid beta-lactams and refer to an allergist after discharge (PGY1: 13%, PGY2: 9%, PGY3: 11%). Delabel the allergy in EPIC and use penicillins (PGY1: 13%, PGY2: 36%, PGY3: 22%). (B) Case 2: A patient with a listed penicillin allergy needs ceftriaxone. What would you do? (p = 0.041, chi-square test). Response: Avoid cephalosporins and choose a different antibiotic (PGY1: 50%, PGY2: 18%, PGY3: 0%). Check the allergy history, and if low risk, proceed with ceftriaxone (PGY1: 50%, PGY2: 82%, PGY3: 78%). Ceftriaxone is ok to prescribe regardless of the severity of penicillin allergy (PGY1: 0%, PGY2: 0%, PGY3: 22%). Order a penicillin skin test or refer to an allergist (PGY1: 0%, PGY2: 0%, PGY3: 0%)

To assess clinical decision-making, residents were presented with two case scenarios. The first case involved a patient with a documented penicillin allergy who reported a mild rash as a child (20 years ago) with no other symptoms (Figure 4A). In the first case, only 39.3% responded correctly: Avoid penicillins before the challenge, but it's ok with some other beta-lactams (p = 0.27). The second case involved a patient with a listed penicillin allergy who required ceftriaxone (Figure 4B). In the second scenario, 54% (n = 15) of respondents correctly stated that they would verify the allergy history and, if the risk was low, proceed with ceftriaxone. A significant difference was observed between interns and senior residents in selecting the correct response (p = 0.041).

## Discussion

Beta-lactams are the most frequently prescribed antibiotics worldwide, which include penicillins, cephalosporins, carbapenems, and monobactams [7-9]. Penicillin allergy is the most commonly documented drug allergy in medical records, with a reported prevalence ranging from 6% to 25% across different populations [7-9]. However, formal evaluation confirms the allergy in only 2% to 10% of reported cases [5,7,8].

According to the Joint Task Force on Practice Parameters Drug Allergy Updates, the primary diagnostic methods for penicillin allergy include skin testing and direct drug challenges [10-12]. While penicillin skin testing is most appropriate for patients with a history of anaphylaxis or recent IgE-mediated reactions, those with remote, benign reactions can be evaluated with a direct oral challenge [10-13]. For patients whose reported penicillin allergies are inconsistent with true allergic reactions, such as those with a history of headache or a family history of penicillin allergy, no testing is necessary, and they can be safely delabeled [10-12]. Although patient histories may be unreliable, risk stratification based on historical features alone has been shown to effectively identify individuals who can safely undergo a direct challenge [12,13]. The PEN-FAST score is the most widely used, safe, and easy tool for the risk stratification of the penicillin allergy, designed to identify those who qualify for the direct oral challenge [14-16]. It was successfully validated by Trubiano et al. and later by Copaescu et al., in the PALACE randomized controlled trial [6,13]. The PEN-FAST score tool consists of four criteria: recent allergy (<5 years, 2 points), history of anaphylaxis or angioedema (2 points), severe cutaneous reaction (2 points), and whether treatment was required (1 point). Total scores classify patients as low-risk (0-1), intermediate-risk (2), or high-risk (≥3). Low-risk patients can often safely undergo a direct oral challenge without prior skin testing, allowing for streamlined delabeling and safer antibiotic use [14-16].

Our study highlights the gaps in knowledge, confidence, and practice patterns among internal medicine residents regarding beta-lactam allergy delabeling. Despite increasing awareness of the clinical and antimicrobial stewardship benefits of removing inaccurate allergy labels, hesitancy remains prevalent.

While a majority (n = 17, 60.7%) of residents reported knowing how to stratify beta-lactam allergy risk, only a small fraction (n = 2, 12%) felt confident in actually doing so. Notably, confidence was lowest among interns, suggesting that the clinical training plays a crucial role in developing competency in allergy assessment. This is further supported by the fact that the self-report of any level of familiarity with tools such as the PEN-FAST score increased with training level (0% among PGY1 vs. 55% in PGY2 vs 77% in PGY3, p = 0.017), yet even among senior residents, formal application of this tool was limited to 22% (n = 2). These findings suggest that while exposure to beta-lactam allergy risk stratification improves over time, hands-on experience and structured guidance remain insufficient.

The significant difference between senior residents (n = 13, 65%) and interns (n = 1, 12.5%) who had previously delabeled a patient (p = 0.048) hints that practical experience in allergy delabeling is acquired progressively, rather than being systematically taught. This finding may also reflect the low number of clinical encounters with progressive accumulation of clinical exposure over time. A concerning finding was that half of the surveyed residents had never attempted to remove an incorrect beta-lactam allergy label, despite that considered to do so. This finding is suggestive of possible barriers that hinder the residents’ actions. The most frequently reported barriers to delabeling were the lack of a clear institutional protocol (n = 23, 82.14%) and insufficient knowledge on how to perform delabeling safely (n = 18, 64.3%). Additionally, time constraints, uncertainty about proper documentation, and concerns about resistance from colleagues further compromised effective delabeling efforts. Addressing these barriers requires institutional support through standardized protocols, workflow integration, and targeted education [17,18].

Our findings suggest that the absence of institutional guidelines and formalized resident training contributes to the persistence of unnecessary allergy labels. To address these issues, a structured, evidence-based allergy delabeling protocol should be developed and integrated into the curriculum for all internal medicine residency training programs. This should include education on validated risk assessment tools such as PEN-FAST, simulation-based training in allergy history-taking, and standardized documentation practices and prompts within the electronic medical record. Additionally, incorporating allergy assessment into antimicrobial stewardship initiatives may help prioritize delabeling efforts in routine patient care. As a next step for this quality improvement (QI) project, our team is developing an initiative focused on implementing a standardized beta-lactam allergy delabeling protocol, incorporating provider education, EMR-based clinical decision support, and periodic audit-feedback cycles to measure outcomes and sustainability.

Our study has several limitations. The sample size was small and was conducted within a single community hospital, which limits the generalizability of our findings. The self-reported nature of the survey introduces a response bias, as residents may have overestimated or underestimated their knowledge and practices. The use of a nonvalidated survey instrument may affect construct validity, as the items have not undergone reliability or validity testing. This study also offers meaningful strengths, including addressing a recognized knowledge gap among internal medicine residents and identifying modifiable institutional barriers, which can inform future educational initiatives and antimicrobial stewardship efforts.

## Conclusions

Our study highlights significant gaps in knowledge, confidence, and clinical decision-making regarding beta-lactam allergy delabeling among internal medicine residents. While exposure to risk stratification improves with training level, hands-on experience, and structured guidance remain insufficient. The absence of clear institutional protocols, uncertainty in documentation, and time constraints might further hinder effective delabeling efforts. To address these challenges, we believe integrating structured allergy delabeling strategies into clinical training and hospital protocols is essential. Standardized education on risk assessment tools, workflow integration within the electronic medical record, and targeted antimicrobial stewardship initiatives may empower providers to make informed decisions.

## Appendices

Quality Improvement Project Survey Questionnaire: Penicillin Allergy Delabeling

1. Which year of residency are you? (multiple choice) ○ PGY1 categorical ○ PGY2 ○ PGY3

2. How often do you review a patient’s allergy history in EPIC before prescribing antibiotics? (multiple choice) ○ Always ○ Most of the time, but sometimes I forget ○ Sometimes, usually, I believe the EPIC will warn me ○ Sometimes, usually, I believe the pharmacist will catch the mistake ○ Comment _________________

3. Do you know how to stratify a patient’s beta-lactam allergy risk (e.g., low-risk vs. high-risk)? (multiple choice) ○ Yes, I feel confident in doing this independently ○ Yes, but I would need guidance ○ No, I am not sure how to do this ○ Why stratify, if you could simply avoid prescribing it? ○ Comment _________________

4. Have you ever delabeled a patient’s penicillin/beta-lactam allergy in EPIC? (multiple choice) ○ Yes, multiple times ○ Yes, once or twice ○ I have considered it, but never acted ○ No, I do not feel comfortable doing so

5. Have you ever heard of the PEN-FAST score? (multiple choice) ○ Yes, I use it in my clinical practice ○ Yes, I abstractly know what it is about, but never used it ○ Never

6. What are the biggest barriers to delabeling beta-lactam allergies in your practice? (select all that apply) (check boxes) □ Lack of knowledge on how to do it safely □ Uncertainty about how to document delabeling in EPIC

□ Lack of clear guidance or institutional protocol □ Time constraints during patient encounters □ Not a priority in clinical workflow □ Concerns about co-resident, attending, or pharmacist pushback □ Other: _________________

7. Quiz: Your patient has a documented penicillin allergy in EPIC, but reports that they had a mild rash as a child (20 years ago) with no other symptoms. How would you proceed? (multiple choice) ○ Avoid beta-lactams and prescribe an alternative ○ Avoid penicillins before a challenge, but ok with some other beta-lactams (e.g., ceftriaxone) ○ Order a penicillin skin test or consult an allergist ○ Avoid beta-lactams and refer to an allergist after discharge ○ Delabel the allergy in EPIC and use penicillins.

8. Quiz: A patient with a listed penicillin allergy needs ceftriaxone. What would you do? (multiple choice) ○ Avoid cephalosporins and choose a different antibiotic ○ Check the allergy history, and if low risk, proceed with ceftriaxone ○ Ceftriaxone is ok to prescribe regardless of the severity of penicillin allergy ○ Order a penicillin skin test or refer to an allergist
